# Endosperm of Angiosperms and Genomic Imprinting

**DOI:** 10.3390/life10070104

**Published:** 2020-07-03

**Authors:** Elizabeth L. Kordyum, Sergei L. Mosyakin

**Affiliations:** Institute of Botany, National Academy of Sciences of Ukraine, 01004 Kyiv, Ukraine; inst@botany.kiev.ua or

**Keywords:** embryo sack, endosperm, genomic imprinting, phylogeny, ploidy

## Abstract

Modern ideas about the role of epigenetic systems in the regulation of gene expression allow us to understand the mechanisms of vital activities in plants, such as genomic imprinting. It is important that genomic imprinting is known first and foremost for the endosperm, which not only provides an embryo with necessary nutrients, but also plays a special biological role in the formation of seeds and fruits. Available data on genomic imprinting in the endosperm have been obtained only for the triploid endosperm in model plants, which develops after double fertilization in a *Polygonum*-type embryo sac, the most common type among angiosperms. Here we provide a brief overview of a wide diversity of embryo sacs and endosperm types and ploidy levels, as well as their distribution in the angiosperm families, positioned according to the Angiosperm Phylogeny Group IV (APG IV) phylogenetic classification. Addition of the new, non-model taxa to study gene imprinting in seed development will extend our knowledge about the epigenetic mechanisms underlying angiosperm fertility.

## 1. Introduction 

The history of science convinces us that scientific thought and discoveries usually develop in a spiral pattern: at new turns, old problems acquire new levels of understanding in the light of new ideas and methodological approaches, as well as new achievements in technology, chemistry, and bioinformatics. A clear example of this is the history of the development of ideas in epigenetics. The term “epigenetics” comes from the Greek word “epigenesis” (epi = on, upon; genesis = origin). It emphasizes the widespread occurrence of the epigenetic mode of inheritance, especially among plants. Features of plant biology provide rich material for discussing the participation of epigenetic systems in gene expression regulation and inheritance in the development and adaptation of plants in ontogenesis, due to their ability to propagate vegetatively by rhizomes, runners, bulbs, tubers, and corms, as well as by plantlets emerging from plant leaves, etc. In addition, annual growth of perennial plants (presumably clones) and apomictic propagation—adventitious embryonia, aposporia, and possibly diplosporia—should be noted as well. Modern ideas regarding the epigenetic systems of regulation of gene expression by DNA methylation/demethylation, modifications of histones and chromatin, RNA interference in plant development (including the phenomena of paramutation, nucleolus domination, genomic imprinting, and gene regulation based on microRNAs), as well as the importance of epigenetic systems in plant protection and resistance against viral infection, are highlighted in numerous articles and comprehensive reviews [1,2,3,4,5].

Genomic imprinting (parent-of-origin-specific gene expression via epigenetics) refers to the epigenetic modification of alleles inherited by the maternal or paternal line, which leads to their different expression depending on the parent genome. Thus, the parental (maternal and paternal) genomes are not functionally equivalent due to genomic imprinting [6]. It seems remarkable that genomic imprinting is now reliably known in plants, in particular dicotyledons and monocotyledons [7,8], first of all, for the endosperm—a highly specialized tissue that not only provides the embryo with necessary nutrients, but also plays a special biological role in the process of formation of seeds and fruits. Although both the embryo and endosperm arise in the same system of the embryo sac after fertilization, they differ in the nature of their reactions to the influence of various factors. Morphophysiological studies of the endosperm in connection with various conditions of its occurrence and development (distant hybridization, pseudogamy, various pollination modes, or its absence) showed the versatility of its functions. Recent investigations have demonstrated that “the endosperm is perhaps the most epigenetically divergent plant tissue, with unique DNA methylation and chromatin structure features” [7]. The existence of highly complex physical and physiological relationships between extra-embryonic tissues, including the endosperm, and the developing embryo has been repeatedly emphasized [9,10,11,12,13,14,15].

It should be noted that the currently available data on genomic imprinting in the endosperm were only obtained in studies of the triploid endosperm formed after double fertilization in the *Polygonum*-type embryo sac, the most common one among angiosperms. So, in this mini-review, we would like to draw attention to the need for research into other types of endosperm, to better understand the essence and peculiarities of molecular mechanisms of seed reproduction in plants, providing biological progress of species, and in evolving biodiversity in general. 

## 2. Genomic Imprinting in the Embryo Sac, Mainly in the Endosperm

For the first time, genomic imprinting, i.e., differential expression of the two alleles of the same gene depending on its parental origin, was described in plants by J.L. Kermicle [16] on the example of a maize-specific gene *R*. In subsequent years, research interest into this phenomenon was renewed after the discovery of *MEDEA*, the first imprinted gene in plants that is essential for their development [17,18]. Later, new imprinted genes have been revealed in the endosperm and great progress has been made in understanding the mechanisms of imprinted gene expression in a mature embryo sac and in the embryo and endosperm after fertilization [6,7,19,20,21,22,23,24,25,26,27]. Interesting parallels have been drawn between the mechanisms underlying genomic imprinting in plants and mammals. Therefore, we have only recently considered the modern ideas on the participation of the epigenetic systems in the regulation of imprinted genes in the embryo sac, mainly in the endosperm, and its functional significance.

The study of DNA methylation in the embryo and endosperm of wild-type *Arabidopsis thaliana* (L.) Heynh. (Brassicaceae/Cruciferae) demonstrated the large-scale changes in methylation accompanying endosperm development and the expression of endosperm-specific genes. Due to certain difficulties involved with the isolation of individual cells of the embryo sac, the hypothesis of differences in the epigenome of the egg and central cell has not yet been fully tested, although the presence of several imprinted genes specific to the endosperm has been proven [28,29,30]. Imprinting is expected to play a role in the epigenetic differentiation of the egg and central cell of an embryo sac before fertilization [6] and then between the zygote and endosperm, as well as between maternal and paternal DNA in the endosperm [7].

It was shown that an allelic copy with reduced or eliminated expression at imprinted loci had higher levels of DNA methylation in the endosperm with the expected constancy [31,32]. Determination of the allele-specific expression of the *MEDEA (MEA*) and *FWA* genes in the embryo and endosperm, carried out on the 6th day after pollination, showed *MEA* mono-allelic expression from the mother-inherited allele both in the embryo and in the endosperm [33]. The expression of maternally inherited *MEA* alleles in the central cell of the embryo sac is activated by the DEMETER (DME) DNA glycosylase, which directly removes the 5-methylcytosine base and is encoded by the *DEMETER* (*DME*) gene [34], which is expressed preferentially in the central cell of an embryo sac before fertilization. It has been shown that the specificity of CG demethylation for maternal sequences is proved by the partial restoration of CG methylation in the endosperm to levels found in other tissues due to *DME* mutations [22,23]. DME also activates the *FWA* gene in the central cell prior to fertilization and is believed to play a common role in the regulation of imprinted genes [32]. Other imprinted genes also have cytosine methylated regions in promoters that are associated with maternally restricted expression. The imprinted expression of many genes, including *MEA, FIS1, FWA*, and other genes, was known to be differentially methylated in a DME-dependent manner [23]. It has been shown that the expression of the *MET1* gene supports the activity of DNA methyltransferase in *A. thaliana*. As in the *met1* mutant, DNA methylation is reduced both in repeating sequences and in a single copy [35]. MET1 has been reported to act as an antagonist to DME in controlling the expression of maternal *MEA* [36].

The main volume of methylation in the endosperm of *A. thaliana* wild type was lower in all sequence contexts, compared with the embryo. In the endosperm, fragments of mobile genetic elements were intensively demethylated, which was accompanied by CHH (H is A, C, or T) hypermethylation of mobile genetic elements into the embryo [21]. In the authors’ opinion, short-term transposon activation in the endosperm is not very important, since its genome is not passed on to the next generation. DNA-dependent RNA polymerase IV (PolIV), which specializes in small RNA-mediated gene silencing pathways, was shown to be highly expressed in the endosperm and its expression is predominantly of maternal origin [37]. It is assumed that activation of transposable elements and, as a result, the production of small interfering RNAs (siRNAs) in the central cell of an embryo sac, which migrate to the egg cell and embryo, could actively contribute to an increase in methylation and silencing of mobile genetic elements in the egg cell and subsequently in the embryo via siRNA transport [20,22,25,27].

An important role in the regulation of imprinted gene expression belongs to Polycomb-group (PcG) proteins, which form a protein complex of about 650 kDa and regulate plant life cycles, usually suppressing the transcription of their target genes [19,28,38,39]. It is assumed that these proteins act at the level of chromatin structure to ensure mitotically inherited repression by H3K27 methylation. In *A. thaliana*, the identified genes encoding PcG proteins were generally named *FERTILIZATION INDEPENDENT SEED (FIS), FERTILIZATION-INDEPENDENT ENDOSPERM (FIE)*, *FIS2*, and *MULTI-COPY SUPPRESSOR OF IRA1 (MSI1)*. The PcG complex regulates maternally and paternally expressed genes [22]. It is assumed that the main function of the FIS PcG complex is the imprinting of paternally inherited genes in the endosperm [19]. A *PHERES1 (PHE1)* target gene was identified that is directly regulated by the FIS PcG protein complex [28,40]. Maternal inheritance of the *FIS2, MEA* (an *FIS* component), and *FIE* genes is necessary for the formation of viable seeds. The FIS PcG complex regulates dosage-sensitive genes in the endosperm [21]. An important component of *MEA* imprinting is repression of the paternal *MEA* allele in the endosperm; this process involves the autoregulation of *MEA* by H3K27 55.68.69 trimethylation [19]. FIE and MEA are proposed to interact directly in wild-type plants to control female gametophyte, endosperm, and embryo development [41]. It was demonstrated that the *PHE1* flanking sequence was demethylated by DME in the central cell, which allows polycomb repressive complex 2 (PRC2) to establish a repressive chromatin environment. *PHE1* imprinting is lost in the endosperm in a DME-dependent manner [23].

The *PHE1* gene encodes a transcription factor of the MADS-box gene family and is usually depressed in the *mea* and *fis* mutants. MADS-box genes are also targeted by some members of the plant PcG [38]. It has been shown that *PHE1* is not expressed in a female gametophyte, but its activity is found in the central cell of an embryo sac in wild-type and *fis* mutants one to two days after pollination. Later in the developing seed, the expression of *PHE1* becomes limited in the chalazal region of the endosperm in the wild type, while in the *mea* and *fis* mutants it remains intense. It has been demonstrated that FIE and MEA proteins are associated with the proximal part of the *PHE1* promoter and the beginning of the coding region [42]. As already noted, *PHE1* is a direct target of FIE and MEA, and, according to the authors, this is the first serious demonstration of direct PcG targets in plants. Partial seed abortion in *mea* mutants is thought to be caused by PHE1 mis-regulation. Ripe seeds were not normal; they were larger, remained green longer, and were more sensitive to dehydration [42]. Currently, investigations of the role of genomic imprinting in endosperm function at the later stages of seed development are being expanded. It was demonstrated that intraspecific variations in allele-specific imprinting of the class IV homeodomain leucine zipper (HD-ZIP) transcription factor *HDG3* in the endosperm is an important determinant of endosperm cellularization (the transfer from the stage of free nuclei to the cellular stage) and seed development phenotypes [43]. Recently, the type I MADS-box transcription factor (TF) PHE1 was reported to be a major regulator of imprinted genes and other genes relevant to endosperm development. PHE1 can establish endosperm-based reproductive barriers in crossing [44]. Regulation of imprinting is suggested to be diverse. The evolutionary and biological significance of genomic imprinting has been emphasized [45].

Very interesting data were reported by P. Khanduri et al. [46] about the disappearance of FIE expression in the flowering stage of *Zeylanidium olivaceum* (Gardner) Engl. and *Polypleurum stylosum* (Wight) J.B. Hall (the latter is now considered to be a synonym of *P. wallichii* (R.Br. ex Griff.) Warm.), both belonging to the family Podostemaceae (order Malpighiales, the rosids clade of eudicot angiosperms), which are submerged aquatic plants growing on rocky substrata under running water, although ZoFIE and PsFIE transcripts were present in the vegetative tissue. In the authors’ opinion, the absence of double fertilization and the endosperm underlie this unique pattern of expression [46]. In the family Podostemaceae, an embryo sac is monosporic and four-celled. A chalazal nucleus degenerates at the two-nucleate stage of female gametophyte development, so the mature embryo sac contains the micropylar egg apparatus–an egg cell and two synergids and a polar nucleus. Double fertilization is absent and the endosperm does not develop. The absence of the endosperm is compensated by the nucellar plasmodium, which is located below the female gametophyte. The nucellar plasmodium begins to form at the stage of the two-nucleate embryo sac by disintegration of cell walls, and it becomes the coenocyte before the fertilization of an egg cell, for example in *Inversodicraea bifurcata* Engl. (now accepted as *Ledermanniella bifurcata* (Engl.) C.Cusset) and “*I. keniensis* sp. nov.” (ined.) Nagendran et Sicolia [47] or during the post-fertilization period, for example in *Tristicha trifaria* Spreng. [48].

## 3. Endosperm

**Figure 1:**
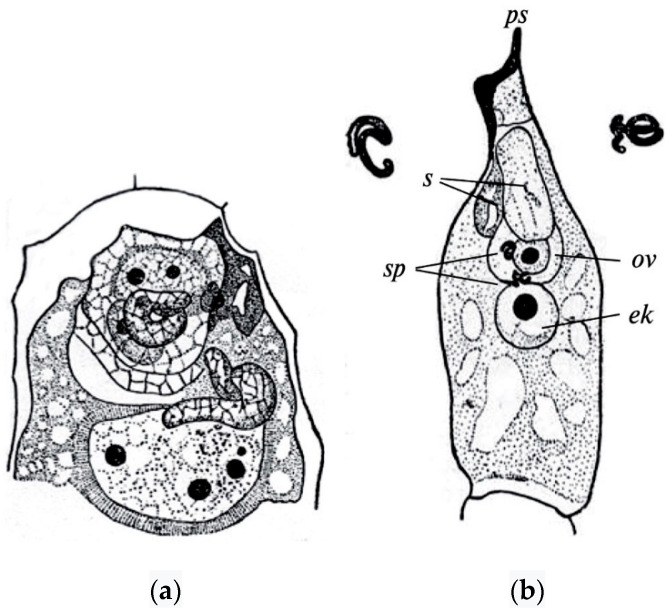
Double fertilization in *Lilium martagon* (**a**) and *Helianthus annuus* (**b**) [67]. ps—pollen tube, s—synergid, ov—egg cell, sp—sperms, ek—nucleus of the central cell of the embryo sac.

The polyploid nature of the endosperm of angiosperms was established due to the discovery of double fertilization in *Lilium martagon* L. by S.G. Navashin [also transliterated as Nawaschin] in 1898 [49]. Of the two sperms that the pollen tube brings to the embryo sac, one sperm fertilizes the egg cell, generating the diploid zygote, whereas the other sperm fuses with the central cell of the embryo sac, giving rise to the endosperm (Figure 1).

Discovery of double fertilization by S.G. Navashin and further demonstration of its presence in almost all angiosperm species, representing diverse evolutionary lineages, showed both the universality and uniqueness of the double fertilization phenomenon for flowering plants [50,51,52,53,54,55,56,57,58]. This has become one of the main arguments in favor of the monophyletic origin of this most highly organized division (mega-diverse, high-rank clade) of the plant world. It has been found that double fertilization is normally carried out in embryo sacs of all types, regardless of the number of polar nuclei fusing with the second sperm nucleus in the central cell of the embryo sac or its ploidy level. In only some species of orchid, for example in tropical *Phaius blumei* Lindl. (now accepted as *P. tankervilleae* (Banks) Blume), the sperm closely contacts with the polar nuclei, but does not fuse with them [51]. The endosperm is not formed in these species of orchids, nor is it formed in Podostemaceae species.

Viable seeds in plants with apomixis develop after fertilization of the polar nuclei in the central cell, a process known as pseudogamous apomixis. Diploid parthenogenesis (diplosporic or aposporic) takes place in such embryo sacs. Aposporic and meiotic embryo sacs can be simultaneously present in the same ovule. In single obligate apomicts, the diploid egg and the tetraploid secondary nucleus in the central cell are able to develop without fertilization [59,60,61]. Numerous experiments on distant hybridization convincingly demonstrated the dependence of embryogenesis on the development of the endosperm. In cases of hybrid endosperm disturbance or its absence, the development of a viable hybrid embryo stops at certain stages and can only be continued if the embryo is isolated and placed on a nutrient medium in vitro (embryo rescue techniques) [10,62,63,64,65,66]. Navashin’s views [67] on the essence and role of double fertilization formed the basis for further ideas about the biological role of the endosperm in the formation of seeds and fruits. It particularly explained the occurrence of xenia in maize, a phenomenon in which kernel characteristics vary as a result of pollination of part of the maize flowers by foreign pollen ([68], p. 724).

### 3.1. Endosperm Types

At present, the formation of three types of endosperm in angiosperms has been clearly established—nuclear, cellular, and helobial.

The nuclear type of endosperm is characterized by the fact that the first division of the primary nucleus of the endosperm (the fusion product of polar nuclei with a sperm) in the central cell of an embryo sac, as well as the division of its derivatives at the initial stages of seed development, result in the formation of free endosperm nuclei, which are located in the cytoplasm layer along the periphery of the embryo sac. The central part of an embryo sac is usually occupied by a vacuole. Generation of cell walls between the free nuclei in different species is carried out with an unequal number of nuclei, ranging from four to eight, or up to a thousand or more. There are variants in the endosperm’s transition from the free-nuclei stage to the cellular stage, including the formation of cell walls from the periphery of an embryo sac to its center, or from the micropylar part to the basal one and vice versa, the degree of filling of the embryo sac cavity with endosperm cells, etc.

In the cellular type of endosperm, the first division of the primary endosperm nucleus in the central cell of the embryo sac and all subsequent divisions are accompanied by cytokinesis. The direction of cytokinesis during the first division of the primary endosperm nucleus can vary. A cell plate is chiefly situated in the transverse direction; a spindle is oriented longitudinally, or less often obliquely. The direction of cell wall laying during subsequent divisions also varies.

In the helobial type of endosperm, the first division of the primary endosperm nucleus in the central cell of an embryo sac is accompanied by cytokinesis, as a result of which there are two cells of unequal volume—the micropylar cell is several times larger than the basal one. In the micropylar cell, free nuclei form first, and the cell walls are laid between them later, as with the nuclear-type endosperm. The chalazal cell functions for the most part as a haustorium—mononuclear, if the cell nucleus no longer divides, or multinuclear as a result of nuclear divisions. In some cases, division of the nucleus in a chalazal cell is accompanied by cytokinesis.

According to the summary provided by G. Davis [52], the nuclear type of endosperm was described in at least 161 families of angiosperms, 83% of which are dicotyledonous (mainly eudicots, sensu Angiosperm Phylogeny Group IV (APG IV) [69]). The cellular endosperm type is characteristic of 79 families, almost all of which, with the exception of Araceae and Lemnaceae (two groups of monocots which are now usually merged in one family, Araceae sensu lato), belong to dicots. The helobial type is known for 17 families, of which 14 are monocots. Although for some families one endosperm type is characteristic, endosperm types vary significantly in other families, often occupying rather distant positions in the phylogenetic system. Combinations of the nuclear and cellular types are more common. For example, this was reported in families such as Alangiaceae (often included in Cornaceae sensu lato), Asclepiadaceae (now usually treated as Apocynaceae subfam. Asclepiadoideae), Asteraceae, Buxaceae, Gentianaceae, Haloragaceae, Hydrophyllaceae (now usually treated as Boraginaceae subfam. Hydrophylloideae), Lauraceae, Nymphaeaceae, Philadelphaceae (now included in Hydrangeaceae), Piperaceae, Rubiaceae, Vacciniaceae (now submerged in Ericaceae sensu lato), and Winteraceae. Nuclear and helobial types of the endosperm were described in families Agavaceae (now usually treated as Asparagaceae subfam. Agavoideae), Alismataceae, Amaryllidaceae, Hypoxidaceae, Linaceae, Spigeliaceae (now included in Loganiaceae sensu lato), Trilliaceae (now included in Melanthiaceae as tribe Parideae). Cellular and helobial types were described in families Olacaceae, Santalaceae, and Thismiaceae, and all three types of endosperm are found in families Boraginaceae and Solanaceae [70]. Species of Podostemaceae and partly Orchidaceae are devoid of double fertilization and endosperms. The subsequent stages of an endosperm’s development—synthesis and accumulation of reserve nutrients, its presence in mature seeds or its resorption during the embryo maturation, etc.—do not reveal any direct connection with its type.

**Figure 2:**
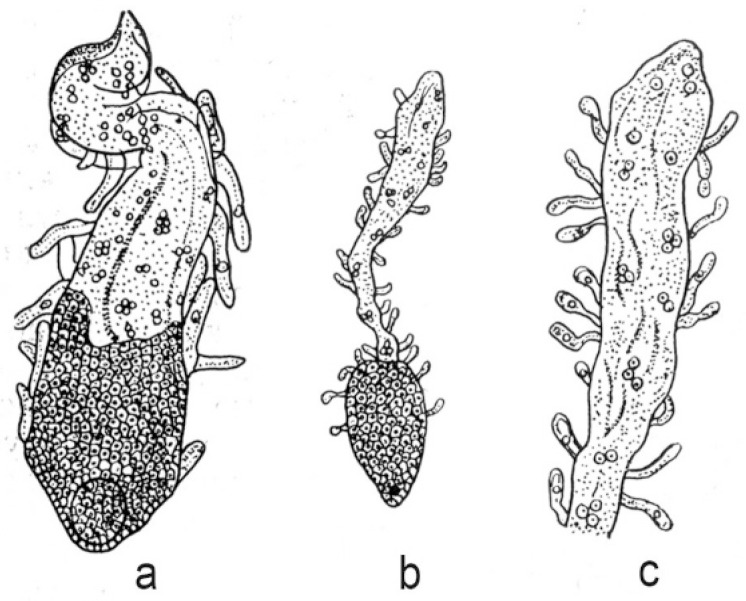
Endosperm and embryo in *Lomatia polymorpha* R.Br. (**a**) and *L. tinctoria* R.Br. (**b**,**c**) [71].

Endospermal haustoria are very diverse in their structure and development, especially in species with cellular and helobial endosperm types. In the nuclear type of endosperm, haustoria arise mainly from the chalazal end of the embryo sac. In most cases they are relatively short and remain at the coenocytic stage. The longest haustoria in embryo sacs with a nuclear type of endosperm are known in the families Euphorbiaceae and Cucurbitaceae. For example, haustoria reach 1000 μm in length in *Croton klotzschianus* (Wight) Thwaites and *C. sparsiflorus* Morong (now usually included in *C. bonplandianus* Baill.). They reach 7398 µm in *Cucurbita pepo* L. and 12,000 μm in *C. ficifolia* Bouché. An interesting feature of nuclear endosperm development has been described in *Lomatia* species of the family Proteaceae [70]. When the embryo consists of 20–30 cells, the endosperm becomes cellular in the micropylar part of an embryo sac; in the chalazal part it remains at the stage of free nuclei. Finger-like unicellular outgrowths appear on the entire surface of the endosperm and their formation increases the absorptive surface (Figure 2).

**Figure 3:**
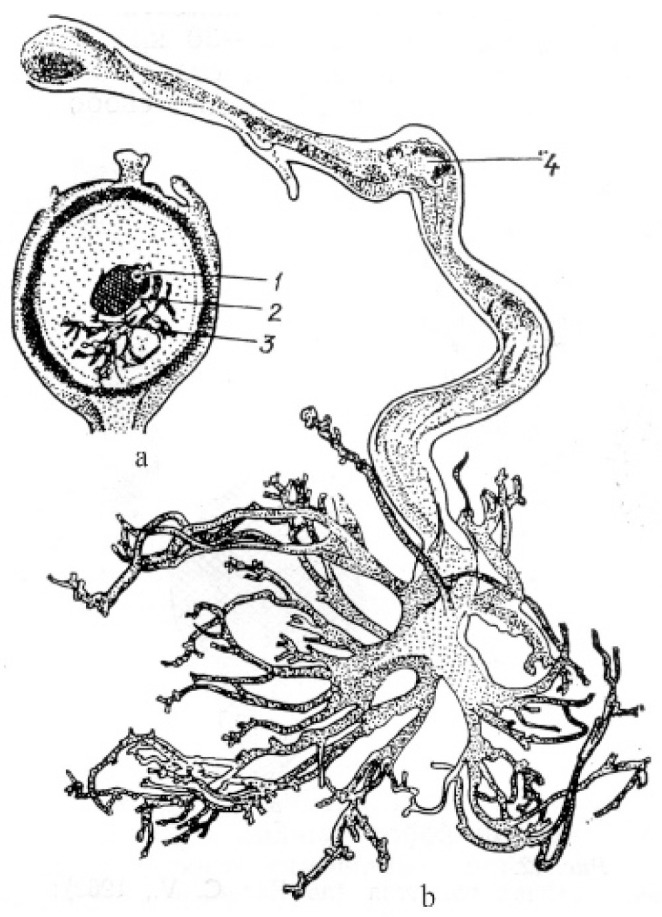
General view of fruit at the stage of a globular embryo (**a**) and haustorium (**b**) in *Jodina rhombifolia* [72]. 1—embryo, 2—endosperm, 3—haustorium, 4—nucleus.

In angiosperms with cellular and helobial types of the endosperm (taxa of families Acanthaceae, Araceae, Boraginaceae, Lamiaceae, Lentibulariaceae, Lobeliaceae, Magnoliaceae, Nymphaeaceae, and Viscaceae), haustoria can arise either from the chalazal end of an embryo sac or from the micropylar end, as well as from both. In *Jodina rhombifolia* (Hook. et Arn.) Reissek (family Santalaceae), a haustorium is formed from a mononuclear chalazal cell [72], and only the micropylar cell gives rise to the endosperm itself. The nucleus of the chalazal cell migrates to the haustorium and greatly hypertrophies. Numerous branches of the free end of the haustorium give it the look of coral (Figure 3). 

**Figure 4:**
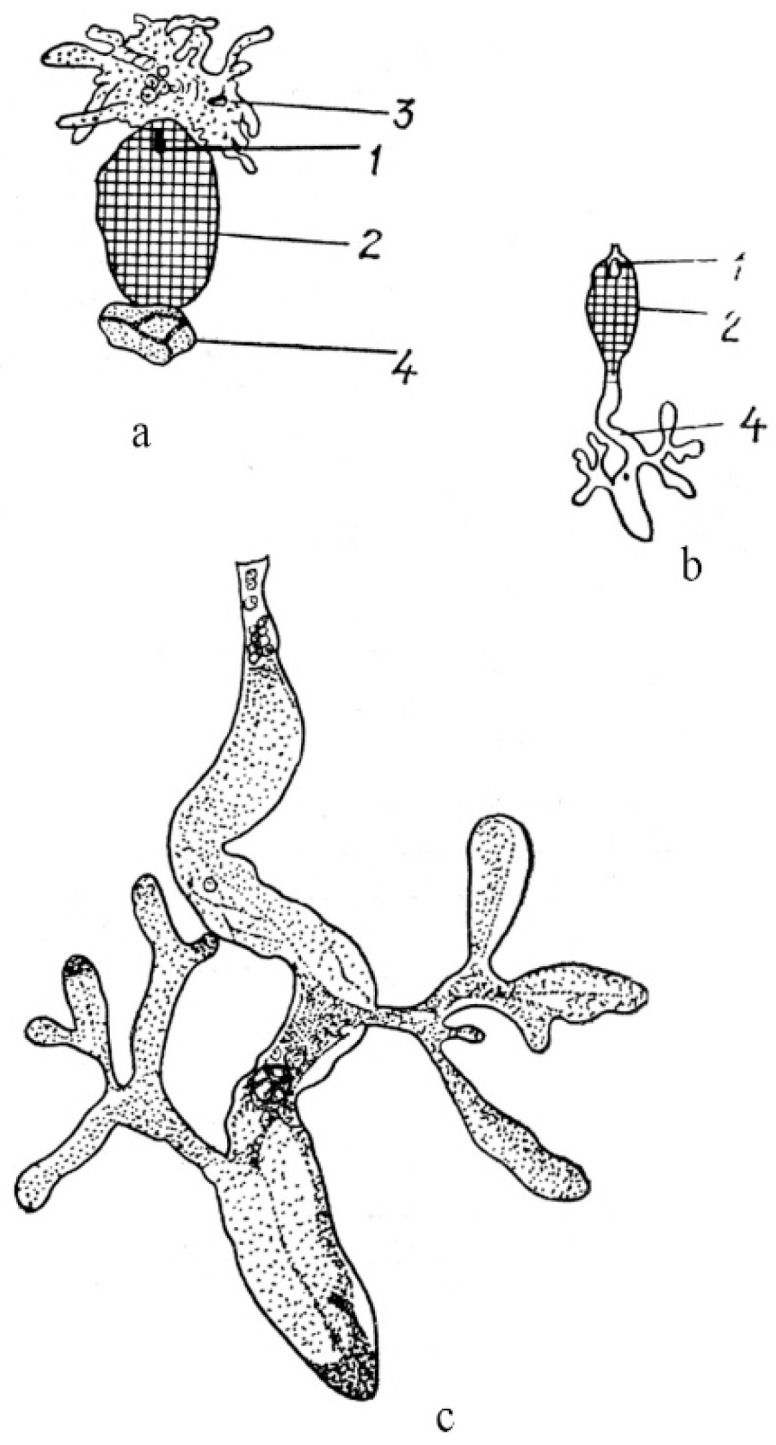
General view of embryo and endosperm with chalazal and micropylar haustoria in *Loasa bergii* (**a**) and with chalazal haustorium in *Blumenbachia silvestris* (= *Caiophora silvestris*) (**b**). (**c**)—fragment of Figure 4b [73]. 1—embryo, 2—endosperm, 3—micropylar haustorium, 4—chalazal haustorium.

Micropylar and chalazal endospermal haustoria are well developed in the family Loasaceae. As reported for *Loasa bergii* Hieronym. (now accepted as *Pinnasa bergii* (Hieron.) Weigend et R. H. Acuña), the chalazal haustorium is spherical in shape and usually contains one hypertrophic nucleus, although haustoria with a large number of nuclei are sometimes observed [73]. The micropylar haustorium contains free nuclei and has hyphoid-like branches reaching significant sizes (Figure 4).

Haustorium branches penetrate the ovule integument and funiculus, sometimes reaching the placenta. The elongated chalazal haustorium in *Blumenbachia silvestris* Poepp. (= *Caiophora silvestris* (Poepp.) Urb. et Gilg) consists of the narrow multicellular base and the highly branched apical part containing only one hypertrophic nucleus. The micropylar haustorium has a similar structure, but it is shorter and wider, and contains numerous nuclei, between which cell walls can form. *Mentzelia laevicaulis* (Douglas ex Hook.) Torr. et A.Gray is characterized by an elongated and unbranched chalazal haustorium consisting of many large cells. The micropylar haustorium is wider and shorter than the chalazal haustorium (Figure 4b,c). In species of the family Scrophulariaceae (as understood in its traditional wide circumscription), in addition to the micropylar and chalazal haustoria, several secondary lateral haustoria can form in the endosperm micropylar and chalazal parts [50,74]. Well-developed and aggressive haustoria of all three types in some species of the family can be formed simultaneously, thus creating a very effective absorptive system.

### 3.2. Embryo Sac Types and Endosperm Ploidy 

Different levels of endosperm ploidy depend on the type of embryo sac, consequently endosperm ploidy levels can vary from 2 *n* (*Oenothera*-type of embryo sac) to 9 *n* (*Peperomia*-type of embryo sac). Therefore, we briefly dwell on the classification of the types of embryo sacs and their distribution over angiosperm families. Of the proposed classifications of types of embryo sacs [75,76,77,78,79], we selected the main types reflecting the evolution of the female gametophyte from the monosporic type to the bi- and tetrasporic types [50,78]. Identification of a type of embryo sac development is based on the following three features: (1) the number of macrospores giving rise to the embryo sac; (2) the number of mitoses that occur during the development of the embryo sac; and (3) the behavior of the nuclei that determines the organization of the embryo sac.

**Figure 5:**
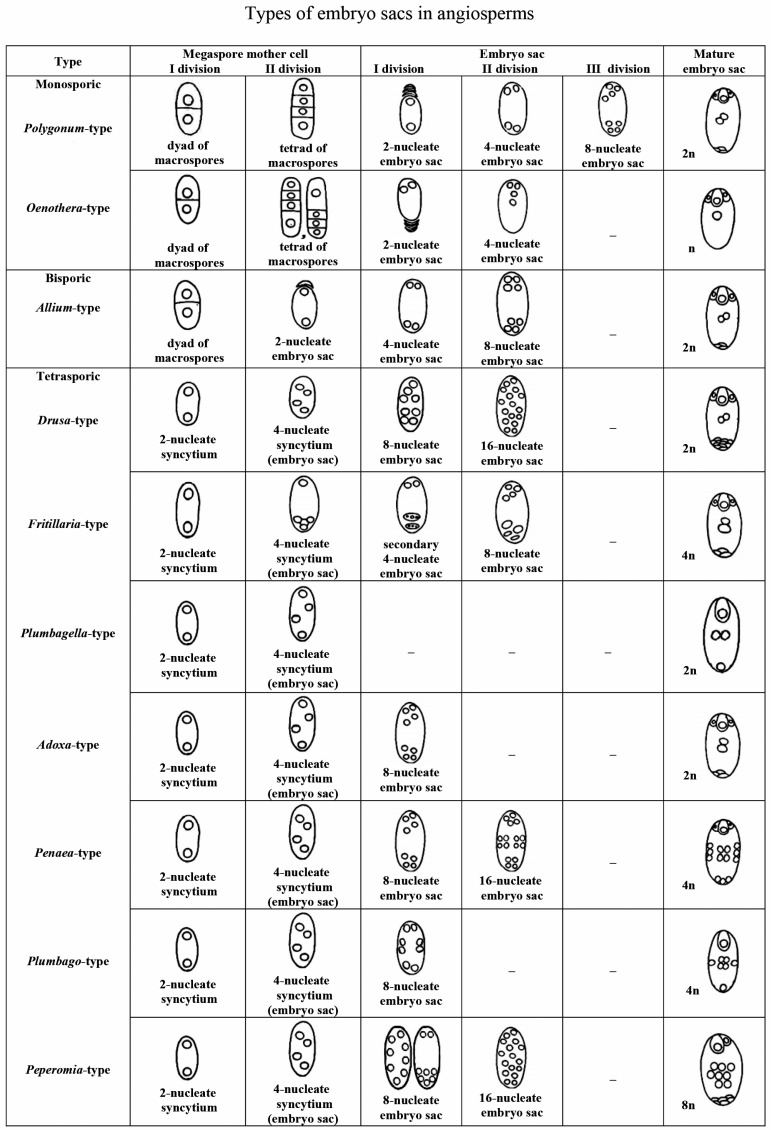
Types of embryo sac development in angiosperms (modified [78]). The number of chromosomes (n) in the secondary nucleus of the central cell of an embryo sac is indicated.

1. *Polygonum*-type (normal type) is single-spore, three-mitosis. The mature embryo sac is characterized by two three-cell polar groups (egg apparatus and antipodes) and two polar nuclei in the central cell. The most variable group is formed by antipodes (rapidly degenerating, normal, hypertrophic, reproducing).
2. *Oenothera*-type: single-spore, two-mitosis. The mature embryo sac has only one polar group: a three-cell egg apparatus and one upper polar nucleus in the central cell.
3. *Allium*-type: bisporic, two-mitosis. The organization of the mature embryo sac is the same as in the normal type.
4. *Drusa*-type: four-spore, two-mitosis. The mature embryo sac contains a three-cell egg apparatus, 11 antipodal cells, and two polar nuclei in the central cell.
5. *Fritillaria*-type: four-spore, two-mitosis. Upon transition from the prophase to the metaphase of the first mitosis, the fusion of three chalazal nuclei occurs. As a result, a secondary four-nucleus embryo sac with a haploid upper and triploid lower pairs of nuclei is formed after the first mitosis. The second mitosis leads to the formation of an embryo sac with the same organization as in the normal type, but with triploid antipodal cells and the lower polar nucleus.
6. *Plumbagella*-type: four-spore. The organization of the mature embryo sac originating directly from the tetranuclear coenocyte is simplified. The egg apparatus consists of only one egg; the central cell contains two polar nuclei and one antipodal cell.
7. *Adoxa*-type: four-spore, single-mitosis. The organization of the mature embryo sac is the same as in the normal type.
8. *Penaea*-type: four-spore, two-mitosis. After two mitoses, four nuclei are formed at each pole. The mature embryo sac contains four polar groups of three cells each (egg apparatus, chalazal, and two lateral) and a central cell with four polar nuclei.
9. *Plumbago*-type: four-spore, single-mitosis. In a four-spore coenocyte, one nucleus is located in the micropylar end, the second is located in the chalazal end, and the other two occupy a lateral position. The nuclei divide, and one cell is formed near each pole. In the mature embryo sac, the micropylar cell is an egg cell. Chalazal and lateral cells usually die. The central cell contains four polar nuclei, which fuse to form a secondary tetraploid nucleus.
10. *Peperomia*-type: four-spore, two-mitosis. After the first mitosis, eight nuclei are distributed usually in the peripheral layer of the cytoplasm or two of them are grouped in the micropylar end, and six are closer to the chalazal end. After the second mitosis, eight nuclei occupy the peripheral position, and the same number of nuclei is in the cell center. In the mature embryo sac there are an egg cell and a synergid in its micropylar end. Six lateral cells are close to the chalazal end of the embryo sac. Eight polar nuclei in the central cell fuse, forming the secondary nucleus (Figure 5).

Among the described variations of the main types of embryo sacs [77,78,79,80] we would like to note the organization of mature embryo sacs in *Tulipa tetraphylla* Regel and taxa of *Tulipa* subgen. *Eriostemones* (Boiss.) Raamsd. reported by I. D. Romanov [79]. In the mature four-spore, single-mitosis embryo sac of *T. tetraphylla*, the egg apparatus consists of five cells, the central cell with two polar nuclei, and the antipodal cell alone with a degenerating nucleus. Cells of the egg apparatus are not differentiated. A mature four-spore, single-mitosis embryo sac of *Tulipa* subg. *Eriostemones* has only one micropylar group: the egg apparatus of seven cells and the upper polar nucleus of the central cell. The egg is not morphologically indistinguishable before fertilization.

The *Polygonum*-type embryo sac has been found in 80% of the examined angiosperm species in 239 families out of about 295 recognized ones. The *Allium*-type embryo sac is characteristic for such families as Alismataceae, Datiscaceae, Malpighiaceae, Theaceae, Limnocharitaceae, Loranthaceae (subfamily Viscoideae, or a separate family Viscaceae of the order Santalales), and Liliaceae. The *Adoxa*-type embryo sac is described in Adoxaceae and Liliaceae families; the *Drusa*-type embryo sac is described in Limnanthaceae; the *Oenothera*-type embryo sac is described in Onagraceae; and *Plumbago*- and *Plumbagella*-types are described in Plumbaginaceae. The *Peperomia*-type is known in the families Euphorbiaceae and Piperaceae; the *Fritillaria*-type in Liliaceae; and the *Penaea*-type in the families Euphorbiaceae, Penaeaceae, Malpighiaceae, and Apiaceae. Of course, those are only some examples of the distribution patterns by families, because particular embryo sac types are much more widespread over the phylogenetic tree of angiosperms.

Only one type of embryo sac is characteristic for a number of families; for example, all species of the family Poaceae studied in this respect, with the exception of apomictic forms, have a *Polygonum*-type embryo sac. In other families and higher-rank groups, at least two different types of embryo sacs are known in one family. For example, the *Polygonum*-, *Allium*- and *Adoxa*-type embryo sacs are described in the families Caprifoliaceae, Caryophyllaceae, Commelinaceae, Orchidaceae, Solanaceae, and Trilliaceae (Melanthiaceae sensu lato); the *Polygonum*-, *Allium*-, *Adoxa*-, *Drusa*-, and *Fritillaria*-type embryo sacs are known in families Asteraceae and Liliaceae; and the *Allium*-, *Adoxa*-, *Drusa*-, *Fritillaria*-, and *Penaea*-type embryo sacs occur in the family Euphorbiaceae [52].

Information on distribution of embryo sac types over the phylogenetic tree of angiosperms is also available from the continuously updated Angiosperm Phylogeny Website; in particular, P.F. Stevens recognizes the following main types: *Adoxa*-, *Allium*-, *Drusa*-, *Endymion*-, *Fritillaria*-, *Oenothera*-, *Penaea*-, *Peperomia*-, *Plumbagella*-, *Plumbago*-, *Polygonum*-, and *Schisandra*-types [81].

We emphasize once again that the trophic and physiological role of the endosperm in the formation of an embryo, seed, and fruit is basically the same, regardless of the type of its development and ploidy, as shown by numerous studies of embryology and biology of the process of seed maturation in a number of cultivated angiosperms in natural and experimental conditions. Attempts to compare particular types of endosperm development with the shape and size of embryo sacs, as well as with a rate of embryo development, have not yielded positive results.

## 4. Embryo Sac and Endosperm Phylogeny

The question of the origin of a female gametophyte of the angiosperms is closely connected with the problem of the origin of angiosperms (however, we do not consider this evergreen and fascinating problem in our article). The peculiarities of the angiosperm female gametophyte structure, which is not found in all other divisions of higher plants, remains unresolved and debated to date. It is sufficient to mention that the current state of botanical knowledge allows us to join the opinion repeatedly expressed in the literature that the emergence of angiosperms was the greatest arogenesis event (a progressive evolution phenomenon, in the terms of A.N. Severtzov [82] and I.I. Schmalhausen [83]) and the evolution of angiosperms proceeded in various directions with a pronounced heterochronism. In the conjugate chain of aromorphoses (roughly corresponding to the modern concept of key evolutionary innovations; see [84] and references therein) of the internal structures of the angiosperm generative organs (the appearance of specific female and male gametophytes, double fertilization and polyploid endosperm, etc.), the final stage is a polyploid endosperm formed as a result of fusion of a sperm cell with the polar nuclei of the central cell of the embryo sac. The currently accepted concept of aromorphosis [82,83] includes the appearance in the body of such progressive changes that are not strictly limited to any particular environment and, thus, raising the organism to a higher level of organization, allowing it to colonize successfully new and often very distinctive and challenging habitats [83]. The attribution of female and male gametophytes in angiosperms to phenomena such as aromorphosis underlines the higher level of their organization as compared to their ancestor and/or co-existing organisms.

Based on the assumption that the embryo sac is a homolog of a female gametophyte of the gymnosperms [85,86,87,88], the archegonial hypothesis of O. Porsch [85] and M. Favre-Duchartre [88], and the gnetaceous hypothesis of F. Fagerlind [86,87] were and still are repeatedly discussed in the literature, but neither of them is universally recognized. J. Coulter [75] expressed the idea of gradual reduction of an archegonium, which begins in gymnosperms and ends in angiosperms with the complete disappearance of its wall, from which only the reproductive structure remains, an egg cell. E. Strasburger (cited in [78]) considered the elements of the angiosperm embryo sac as the initial stages of the female gametophyte development, practically before the formation of archegonia, like in *Gnetum*. Currently available data on the comparative morphology and embryology of *Gnetum* species do not give reasons to consider the organization of the female gametophyte of Gnetales as basic for an embryo sac of angiosperms, but rather as a variant of the female gametophyte from the distant past which has been preserved, which has not received further development, and which currently represents a kind of “evolutionary dead end”. A clearly formulated hypothesis of the neotenic origin of the embryo sac was proposed by I.D. Romanov [79]. Differentiation of an egg cell occurs at the very early stages of the female gametophyte development, at the latest after the third division of a nucleus of the macrospore, which naturally excludes the formation of the archegonium. E. N. Gerasimova-Navashina [89] further developed these views and also considered the embryo sac as a greatly reduced female gametophyte of some earlier ancestral forms. In her opinion, general laws that govern the development and organization of any cell being in certain conditions are manifested in the evolution of an embryo sac.

The prevailing point of view is that the *Polygonum*-type embryo sac (monosporic, three-mitosis, eight-nucleate, seven-celled) is the initial or ancestral one, whereas the other types, which are different variations of the main type, are likely derivatives. Less common is the belief that other types of embryo sacs—for example, a four-spore 16-nucleate embryo sac—are more primitive or evolutionarily independent from the *Polygonum*-type. According to Modilevsky [51], mono-, bi- and tetrasporic embryo sacs have a common origin, but from the very first days of their appearance they differed by the patterns of formation, thus being peculiar and equivalent variants of one general type. This assumption is based on the presence of a four-spore female gametophyte in *Gnetum gnemon* L. (*G. ovalifolium* Poir.), as well as on the formation of 16-nucleate embryo sacs in a number of angiosperm families, for example Euphorbiaceae, among the *Polygonum*-type embryo sacs typical of these families. It should be noted that formation of mono-, bi- and tetrasporic embryo sacs with the dominance of a bisporic embryo sac can occur in different ovules of the same species, for example in *Erigeron elatus* Greene.

**Figure 6:**
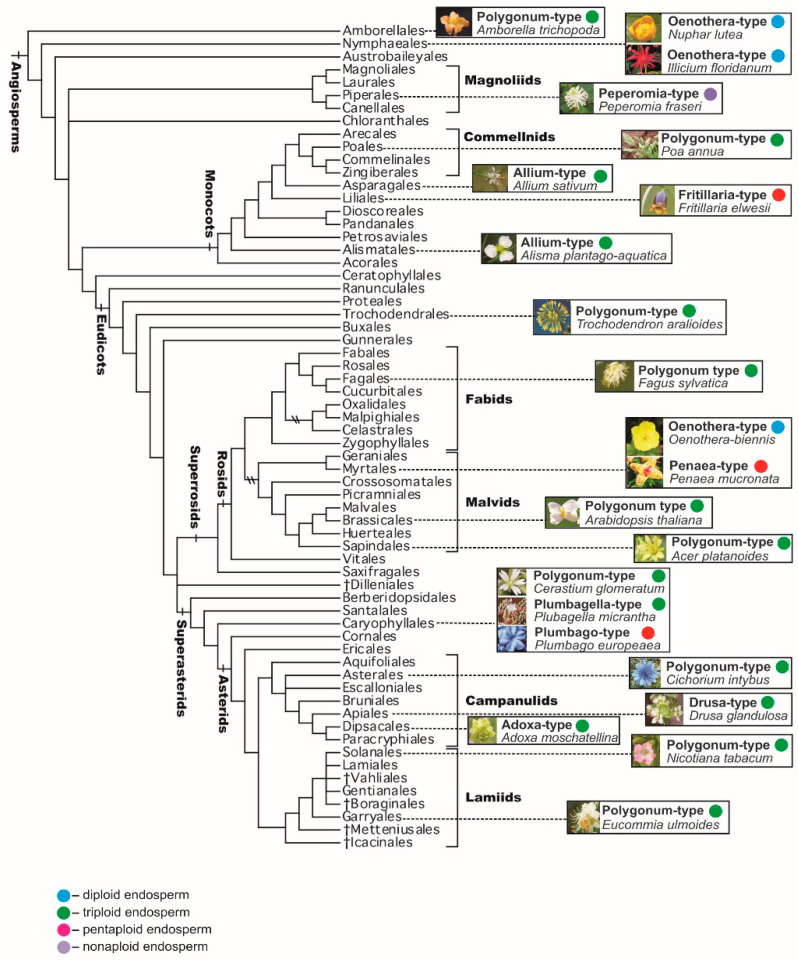
A schematic representation (selected examples only) of the distribution of the main types of embryo sacs and, consequently, endosperm ploidy levels, through a phylogenetic tree based on Angiosperm Phylogeny Group IV (APG IV, 2016) [69]. Further details and examples are provided in the text.

It should be also emphasized that the *Polygonum*-type is present in several plant families that occupy especially important positions on the phylogenetic tree of angiosperms [69,81,90,91]. In particular, the *Polygonum*-type is typical for (1) the family Amborellaceae, representing the basalmost (the most early-branching) angiosperm clade sister to the clade containing all other angiosperms; (2) other representatives of the ANA grade (orders Amborellales, Nymphaeales, and Austrobaileyales), representing the basal grade of angiosperms, although in Amborellales the embryo sac is 9-nucleate, whereas in the two other orders it is 4-nucleate; (3) the family Acoraceae (with the single genus *Acorus*), which forms the basalmost clade of monocots; and (4) in early-branching eudicots, such as representatives of the family Ceratophyllaceae and most taxa of the order Ranunculales (families Lardizabalaceae, Circaeasteraceae, Menispermaceae, Berberidaceae, and Ranunculaceae; in the last family the *Allium*-type is also known). Such predominance of the *Polygonum*-type in basalmost or early-branching clades of angiosperms in general, and their main clades in particular, indicates that that type was probably the ancestral one (Figure 6).

Discussion of the evolution and homology of the endosperm of angiosperms is mainly based on two alternative hypotheses. These hypotheses have been actively discussed in the past and have received new developments in connection with data from a cladistic analysis of the basal species of angiosperms, as well as from cases of fertilization of female cells with a second sperm in species of *Ephedra* and *Gnetum* [92,93,94,95,96,97,98,99]: (1) the endosperm is homologous to the embryo, i.e., it can arise from the transformation of the additional embryo development as a result of the second act of fertilization, which first occurred in the ancestors of the angiosperms; and (2) the endosperm is homologous to the female gametophyte, as a product of altered ontogenesis of female gametophytes of non-flowering seed plants, which later sexualized. In addition, a triploid endosperm could be considered homologous to the gymnosperm female gametophyte in the evolutionary context of the parental conflict hypothesis [100,101,102].

Two cases of penetration of two sperms from the pollen tube into the cell central of an archegonium in *Ephedra nevadensis* S. Watson and *E. trifurca* Torr. ex S. Watson were described [92,93]. One sperm fused with the egg cell, and the second with the abdominal canal nucleus, resulting in the formation of two zygotes and, as a consequence, the additional embryo. According to W. E. Friedman [94], this fact may support the point of view that the transitional stage in endosperm evolution was the formation of additional embryos with modified endosperm function to improve the developmental conditions of the sister embryo. In *G. gnemon*, which is characterized by the absence of archegonia [103], the fusion of each of two sperms with individual female cells in the micropylar part of the female gametophyte also described [97]. Based on these observations, the hypothesis about the origin of the gymnosperms and angiosperms from a common ancestor was revived [104,105,106]. Recognition of the potential homology of the reproductive traits of Gnetales and angiosperms is a key point in the hypothesis on the origin of double fertilization from their common ancestor. However, the results of modern molecular phylogenetic studies, as well as earlier morphological studies (as already mentioned), do not support these ideas, but indicate that Gnetales are a monophyletic group closely related to conifers [107,108]. It should also be noted that formation of a second zygote is a result of additional fertilization in Gnetales. Both zygotes give rise to identical multicellular embryos, which can be considered a phenomenon of polyembryony.

Features of the origin and genetic constitution of the endosperm of gymnosperms and angiosperms do not give reasons to consider them homologous formations, although the function of the latter after fertilization is the same. In gymnosperms, the endosperm is a vegetative cell of the female gametophyte, the development of which begins with the germination of macrospores. It is basically composed of haploid cells. The development of the endosperm of angiosperms occurs, as a rule, only after triple fusion, i.e., the fusion of sperm with the polar nuclei of the central cell of an embryo sac, resulting in the formation of the primary nucleus of the endosperm.

The accumulation of reserve substances in the endosperm of gymnosperms begins, as in angiosperms, during embryogenesis. Therefore, we can clearly observe similarity between the endosperm of angiosperm plants and the female gametophyte of gymnosperms in the process of seed maturation, but not as any evidence of their homology. Moreover, convergence takes place mainly at the level of tissues and cells that are functionally similar.

On the basis of a cladistic analysis, the possible primacy of a diploid endosperm, which is formed after fusion of a sperm with a haploid nucleus in the central cell of four-celled embryo sacs in species of *Nuphar* (Nymphaeaceae) and *Illicium* (Illiciaceae), both belonging to basal angiosperms, is discussed [98]. Based on these data, the four-celled female gametophyte, and thus the diploid endosperm, can be considered ancestral to the seven-celled female gametophyte and triploid endosperm [109]. At the same time, a monosporic seven-celled embryo sac of *Polygonum*-type, characteristic of most species of angiosperms, was found in the basalmost angiosperm *Amborella trichopoda* Baill. (Amborellaceae) [110]. We can add that the monosporic four-celled embryo sac, described in species of *Nuphar* and *Illicium*, is typical of the *Oenothera*-type of embryo sac inherent to the family Onagraceae (Figure 6).

Currently, the possibility of a twofold occurrence of diploid endosperm from the initial triploid one in different clades is considered. Phylogenetic patterns of the emergence of the triploid endosperm, once in *Amborella* with a seven-cell embryo sac and once in a common ancestor of monocots, magnoliids, and eudicots, is widely discussed [111]. The triploid endosperm is also assumed to emerge only once in the whole evolutionary history of flowering plants, if the *Polygonum*-type embryo sac is recognized as being peculiar to the last common ancestor of all angiosperms. At the same time, the occurrence of bisporic and tetrasporic embryo sacs and, consequently, higher-ploidy endosperms could have emerged more than once, or even many times [80].

Thus, the question of the origin of the female gametophyte and endosperm of the angiosperms remains open, and numerous hypotheses about the initial or ancestral type of the embryo sac and endosperm, and their derivatives in the extant angiosperms, are largely debatable. The main difficulty in covering these issues lies primarily in the absence of paleobotanical data concerning the various stages of the formation of generative organs in higher plants. Therefore, in assessing the phylogenetic value of traits, subjective elements depending both on the state of scientific knowledge in a certain period and on the views of a researcher will inevitably manifest themselves. Concerns were expressed about the existence of a “vicious circle” in assessing the primitiveness or progressiveness of morphological (in the broad sense of the word) traits: certain traits are considered to be an indicator of primitive initial organization, depending on the position of the organism in a phylogenetic system, yet at the same time, the position of the organism in the currently accepted system is determined by these same traits. Cladistics and molecular phylogenetic approaches do not eliminate completely the existing difficulties, although they allow for the discussion of the “old” problems from new, much improved positions. Broadening the boundaries of knowledge and confirming or denying what has been achieved inevitably raises new discussion issues in such a complex problem as the history and evolution of the unique features of the generative organs in angiosperms.

## 5. Concluding Remarks

Current research on genomic imprinting in the endosperm has considerably expanded our understanding of its biological role in seed and fruit development, and has also created new possibilities to study molecular and genetic mechanisms involved in endosperm formation and functioning. However, it should be noted that available data have been obtained only in studies of the triploid endosperm formed after double fertilization in the *Polygonum*-type embryo sac, the most common type among angiosperms.

Summing up the research on gene imprinting in the endosperm, M. Gehring and P.R. Satyaki [7] outlined most topical questions which are still outstanding. Among them, we would like to emphasize the issues related to “the exact mechanisms by which imprinted genes and *genome dosage* regulate endosperm cellularization timing”. The wide spectrum of endosperm types and ploidy levels pose great opportunities to solve these questions and to analyze the other parent-of-origin effects. A ratio of paternal and maternal genetic information after double fertilization, i.e., “gene dosage” and subsequent transcript abundance in the endosperm must change significantly as its ploidy alters. Variations of the endosperm ploidy from the 3n to 2n (embryo sac of *Oenothera*-type) and on the contrary to 5n (embryo sac of *Plumbago*-type) and 9n (embryo sac of *Peperomia*-type) can be the excellent objects for testing the parental (kinship) conflict model [93] and a dosage-sensitive regulatory model [112,113]. Using such objects also provides new approaches to understanding the complex relations of the endosperm with the embryo, in particular the possible “defense” role of the endosperm in the silencing of transposons in the embryo via transport of siRNA formed as a result of intensive demethylation of mobile genetic elements in the endosperm after fertilization. In addition, it is of interest to study the DNA methylation/demethylation of imprinted genes in aposporic and diplosporic embryo sacs after fertilization of only the polar nuclei (pseudogamy), i.e., the relations of a parthenogenetic embryo (maternal origin) and endosperm (maternal and paternal origin). The comparative investigations of spatial nuclear organization in endosperms of nuclear, cellular and helobial types at the successive stages of their development, including the formation of endospermal haustoria, could be useful in answering the question of how the spatial nuclear organization of endosperm chromatin relates to gene imprinting [7]. Understanding the role of genomic imprinting in post-zygotic incompatibility associated with abnormal development of the endosperm under self-fertilization of cross-pollinated plants and distant hybridization, i.e., interspecific crosses, may be continued in studies of wild species with endosperms of various ploidy levels. Finally, an answer may be obtained to the question of whether the ploidy of endosperms above 3 n is an adaptive advantage or an “excess” of development.

In general, the addition of new non-model taxa to study gene imprinting in the embryo sac before and especially after fertilization will extend our knowledge about the parent-of-origin effects in seed development in angiosperm species occupying different places in the system and the epigenetic mechanisms underlying plant seed reproduction on the whole.
